# French adaptation and validation of the Niigata PPPD Questionnaire: measure of severity of Persistent Postural-Perceptual Dizziness and its association with psychiatric comorbidities and perceived handicap

**DOI:** 10.3389/fneur.2024.1388805

**Published:** 2024-07-30

**Authors:** Vasiliki Meletaki, Maélis Gobinet, Jacques Léonard, Maya Elzière, Christophe Lopez

**Affiliations:** ^1^Aix Marseille Univ, CNRS, Centre de Recherche en Psychologie et Neurosciences (CRPN), Marseille, France; ^2^Centre des Vertiges, European Hospital, Marseille, France

**Keywords:** vestibular system, dizziness, anxiety, depression, mental health, Persistent Postural-Perceptual Dizziness (PPPD)

## Abstract

Persistent Postural-Perceptual Dizziness (PPPD) is a functional vestibular condition. Despite being the most common chronic neuro-otologic disorder, it remains undertreated. The Niigata PPPD Questionnaire (NPQ), developed by Yagi et al. in 2019 to assess the severity of PPPD, could be a useful tool to help in the screening and diagnosis of this condition. This study aimed to validate a French version of the NPQ and make it an available assessment tool. Moreover, we aimed to understand the characteristics of PPPD patients better. The NPQ was translated and adapted into French. 50 PPPD patients, 50 patients with vestibular disorders without PPPD, and 50 healthy controls were included. They answered the adapted NPQ and additional questionnaires assessing trait (STAI) and state anxiety (HADS-A), depression (HADS-D) and handicap related to dizziness (DHI). The NPQ’s reliability was assessed by Cronbach’s alpha. Intergroup comparisons and multiple linear regressions were conducted to examine the characteristics of PPPD patients compared to vestibular patients and healthy controls, to validate NPQ’s reliability, and to explore the effect of clinical parameters and treatment with selective serotonin reuptake inhibitors. Receiver operating characteristic (ROC) curves were carried out to determine the diagnostic values of the NPQ total score and sub-scores. Relations between NPQ and reported handicap, depression and anxiety were evaluated by correlations between questionnaire scores. The internal consistency was high (>0.8) for all NPQ subscales and the total score. Intergroup comparisons showed a significantly higher NPQ total score and sub-scores in the PPPD group compared to the two others. The ROC curve analysis showed a significant, but poor, discrimination of NPQ (AUC = 0.664) and its subscales. DHI scores, depressive symptoms and trait anxiety were significantly higher in PPPD patients than in vestibular patients and healthy controls. State anxiety did not differ between patients with PPPD and vestibular patients without PPPD. Finally, there was a significant correlation between the NPQ and the DHI. Our study provides a better understanding of PPPD symptomatology and its assessment. It showed that the NPQ is a reliable tool that can assist in symptom assessment for a French-speaking population.

## Introduction

Vestibular disorders, often manifesting as vertigo, dizziness, and postural imbalance, impact significantly an individual’s quality of life. Among these conditions, Persistent Postural-Perceptual Dizziness (PPPD) is a distinct and challenging entity with a complex interplay of sensory and perceptual dysfunctions, and psychological factors, that lead to functional alterations of neurologic systems involved in postural control and locomotion (1). PPPD is a relatively recent diagnostic term introduced to encompass a subgroup of patients with functional disorders previously labeled as phobic postural vertigo, visual vertigo, space motion discomfort or chronic subjective dizziness (2). The diagnostic criteria for PPPD, as outlined by the International Classification of Vestibular Disorders, include the presence of persistent non-spinning dizziness lasting 3 months or more, exacerbated by upright posture, active or passive movements, and exposure to moving or complex visual stimuli (2).

PPPD diagnosis is challenging due to similarities and co-existence of other vestibular and psychological conditions. The diagnostic assessment involves a thorough clinical history, physical examination, and exclusion of other potential causes of dizziness and often requires collaboration between neurologists, otolaryngologists, and mental health professionals (1). Despite its prevalence and impact on daily life, people with PPPD often wait for years for a proper diagnosis and treatment course (2–4). PPPD is more prevalent in individuals around 30 to 50 years old, and females (5, 6). The lack of specific biomarkers and the subjective nature of symptoms contribute to the complexity of accurate diagnosis (6–8). In recent years, efforts have been made to develop more standardized diagnostic tools and criteria to enhance the reliability and consistency of PPPD diagnosis. One of them is the Niigata PPPD Questionnaire (NPQ) developed by Yagi et al. (9), which we aimed to translate and validate in a European French-speaking population.

Current treatments for PPPD often involve a multidisciplinary approach, combining vestibular rehabilitation, cognitive-behavioral therapy, and medication aimed at alleviating symptoms and improving patients’ overall well-being. The most used medications are serotonin reuptake inhibitors (SSRIs), which seem to be effective on the dizziness-related handicap (1). Due to the complexity and the subtlety of the symptoms, challenges persist in raising awareness among healthcare providers and providing diagnostic tools, highlighting the need for continued research and tool validation to enhance the assessment and management of PPPD symptoms.

The NPQ, based on the criteria from the Bárány Society (2), can help in the diagnosis and assessment of PPPD. This questionnaire was validated in patients suffering from PPPD and a control patient group with other vestibular disorders in Japan. The NPQ has been used to assess subjective symptoms of patients with PPPD and other vestibular disorders (10), and as a predictor of development of PPPD (7, 11). The NPQ has already been translated and culturally adapted to Spanish (5) and German (12). However, there is no French version of the NPQ available, even though around 30% of the French-speaking population worldwide, i.e., over 200 million individuals, might experience vertigo at some point in their lives.

The current study presents the translation and adaptation of the NPQ in French. We aimed to better understand the characteristics of vestibular and PPPD patients in France and provide a validation of the NPQ as a tool to assist clinicians in the diagnosis and assessment of symptom severity. To that end, we collected information on dizziness handicap, anxiety, and depression, as well as clinical and sociodemographic data to relate them to NPQ scores. As Yagi et al. (9) discussed a potential confounding effect of pharmacological treatments on the NPQ scores, and SSRIs are the most studied pharmacological treatment for PPPD (1, 3), we decided to explore their effect on the NPQ scores. Thus, we also collected data on the patient’s pharmacological treatment at the time of inclusion in the study. We predicted that PPPD patients would have higher scores in the NPQ and perceived dizziness handicap in comparison to other vestibular populations. Secondly, we predicted that vestibular and PPPD patients would exhibit higher scores in depressive symptoms and anxiety than the control population with no vestibular or other psychiatric or neurological disorders. Finally, we predicted that the symptom severity of PPPD (as measured by the NPQ scores) would be positively correlated with anxiety, depression, and the severity of the perceived handicap.

## Materials and methods

### Participants

**Figure 1:**
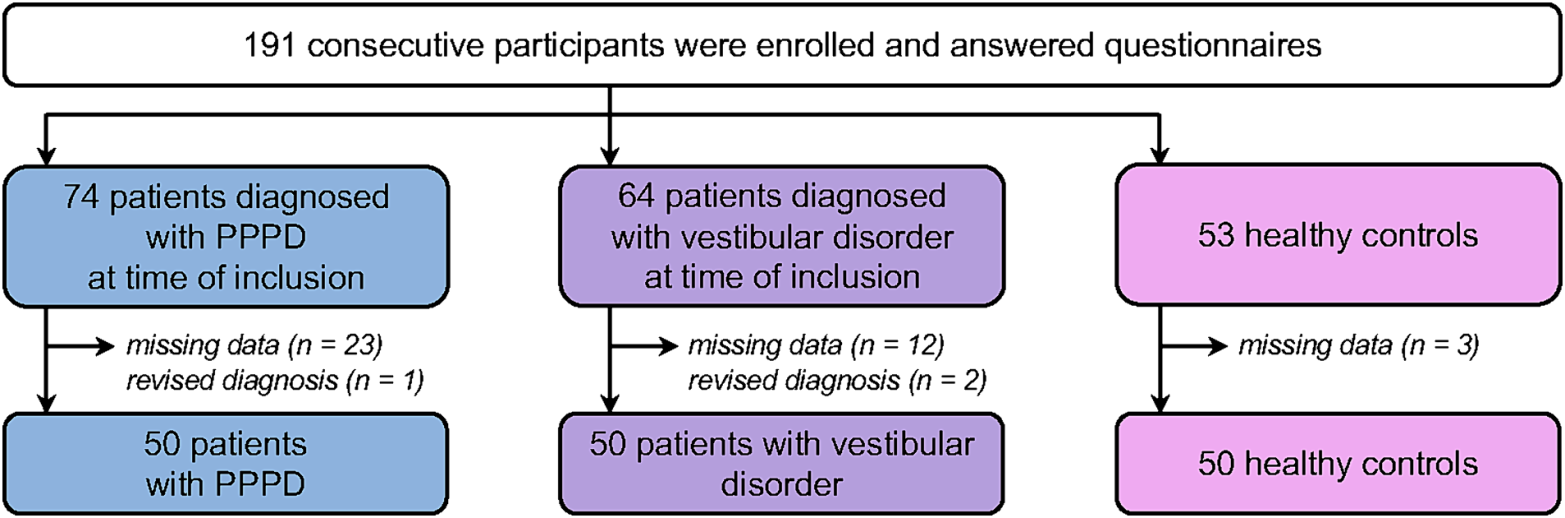
Flowchart of patient’s inclusion.

We aim to validate the French translation and adaptation of the NPQ in a sample of participants similar to that used in Yagi et al. (9) for the validation of the original NPQ. Therefore, we aimed to include 50 patients diagnosed with PPPD according to the criteria from the Bárány Society (2). In line with Yagi et al. (9), we also aimed to include 50 patients with vertigo and dizziness other than PPPD. As PPPD traits may manifest as a continuum in the general population (13), we aimed to include 50 control participants who reported no history of otoneurological disorder to document the NPQ scores distribution in the general population. Figure 1 shows the flowchart of patients’ inclusion. 191 participants completed the questionnaire, of which 41 were excluded because of multiple missing data due to technical issues with the software recording the participant’s responses (*n* = 38), or because the initial diagnosis was revised (*n* = 3). Final analyses included 50 participants per group. Patients with PPPD and patients with vestibular disorders were patients referred to our otoneurological center and diagnosed by our otoneurologist. Control participants were patient’s relatives and hospital staff, who were matched prospectively on age and sex to both clinical groups.

Otoneurological assessment included a battery of standard tests which depended on the patient’s symptoms. The assessment included examination of spontaneous and positional nystagmus, as well as examination of nystagmus evoked by the head shaking test, further investigated by videonystagmography (Synapsys®), the pendular rotatory test (Earth vertical axis rotation on a motorized chair following sinusoidal rotation; Synapsys®), and the caloric test (irrigation of ears with warm and cold water; Synapsys®), examination of the vestibulo-ocular reflex using the video head impulse test (vHIT; Otometrics®), a measure of the amplitude and latency of the cervical vestibular-evoked myogenic potentials evoked by air-conducted sounds on the sternocleidomastoid muscles (Interacoustics®), audiometry (Interacoustics®), and/or MRI with contrast injection. These clinical tests were in general not performed at the time of the NPQ assessment and were taken from the patients’ files. We decided to use these elements as objective indicators of vestibular disorder in the patients’ medical history. Control participants received no otoneurological assessment.

### Ethical approval and patient’s consent

Ethical approval was granted by the Ethics Committee (CPP Île de France 2, n° 2021-A03111-40) and procedures were in accordance with the Declaration of Helsinki. All participants gave their written consent before being involved in the study. They received no payment or compensation.

### French translation and cross-cultural adaptation of the NPQ

The NPQ contains 12 items evaluating difficulties in daily life when facing three main categories of exacerbating factors (9): 4 items pertain to the factor “Upright posture/Walking” (e.g., *Walking at a natural pace*), 4 items pertain to the factor “Movement” (e.g., *Riding a car, bus, or train*), and 4 items pertain to the factor “Visual stimulation” (e.g., *Watching TV or movies with intense movement*). Answers to each item are rated from 0 (“None”) to 6 (“Unbearable”). Thus, each of the three subscales has scores ranging from 0 to 24, and the NPQ total score ranges from 0 to 72. For the NPQ total score, a cut-off score of 27 has been proposed (9).

The rights to translate the NPQ into French and to reproduce it were granted by Wolters Kluwer Health, Inc., the publisher. The steps for the French translation and cross-cultural adaptation are summarized in Supplementary material 1 and were as follows:

1. *Forward translation*. A bilingual French translator, with French as the native language and familiar with otoneurology and vestibular medicine, translated the instructions and questionnaire items into French.
2. *Back translation*. The forward-translated questionnaire was translated back to English by a bilingual English translator, with English as the native language, who was naive to the NPQ and the study purpose.
3. *Back translation review*. The French and English translated questionnaires were compared with the original version of the NPQ (9) to check for congruency. The questionnaire was corrected accordingly to obtain the final questionnaire reproduced in Supplementary material 2.

### Additional questionnaires

#### Dizziness handicap inventory

Handicap related to dizziness was measured with the French adaptation of the Dizziness Handicap Inventory (DHI) (14). This self-reported scale includes 25 items assessing the impact of dizziness on a patient’s quality of life through three factors (emotional, functional, and physical). Each item can be answered by “no” (0), “sometimes” (2) or “yes” (4). The total score ranges from 0 to 100. The French version of the DHI has correct psychometric properties (15).

#### Hospital anxiety and depression scale

States of depression and anxiety were measured using the Hospital Anxiety and Depression Scale (HADS) (16). The HADS is a 14-item self-reported scale with 7 items tapping into anxiety (e.g., *Worrying thoughts go through my mind*) and 7 items tapping into depression (e.g., *I feel as if I am slowed down*). Answers to each item are rated from 0 to 3, contributing to an *anxiety subscale* (HADS-A) and a *depression subscale* (HADS-D). Each subscale has scores ranging from 0 to 21. The HADS shows high sensitivity and reliability in assessing the symptom severity of anxiety disorders and depression in various clinical populations, as well as in the general population (17). The validated French version of the HADS (18) has good internal consistency, similar to other countries.

#### State–trait anxiety inventory

Trait anxiety was measured by the Trait Form (Y-2) of the State Trait Anxiety Inventory (STAI) (19). The STAI Form Y-2 is a self-reported measure of 20 items. Each item can be rated on a scale ranging from 1 (almost never) to 4 (almost always), and the total score can range from 20 to 80. A high score is linked to a high level of trait anxiety. The STAI has been found to have good reliability and validity in clinical populations and the general population (20), as is the case for its French version (21, 22).

#### Demographics

Basic demographic information was collected, including age, sex, handedness, education level, marital status, and employment status. We also collected information about smoking and drinking habits, history of migraine, and antidepressant treatment (Table 1).

**Table 1:** Demographic and clinical characteristics of participants.

	PPPD	Other vestibular disorders	Healthy controls	Statistics^*^
	(*n* = 50)	(*n* = 50)	(*n* = 50)	
Age (mean ± SD), years	49.2 ± 14.2	51.9 ± 12.8	49.1 ± 13.8	*F*(2) = 0.68, *p* = 0.509
Females/Males (*n*, %)	39/11 (78/22%)	38/12 (76/24%)	32/18 (64/36%)	*χ*^2^(2) = 2.89, *p* = 0.236
Right/Left Handedness (n, %)	43/7 (86/14%)	44/5 (90/10%)	47/3 (94/6%)	*χ*^2^(2) = 1.77, *p* = 0.413
Highest education level (%)				*χ*^2^(8) = 7.52, *p* = 0.482
*Level 1*	14%	30%	16%	
*Level 2*	27%	12%	20.5%	
*Level 3*	23%	24%	29%	
*Level 4*	18%	12%	14%	
*Level 5*	18%	22%	20.5%	
Employment status (%)				*χ*^2^(6) = 11.39, *p* = 0.077
*Employed*	70%	68%	59%	
*Student*	2%	0%	14%	
*Retired*	18%	24%	18%	
*Unemployed*	10%	8%	9%	
Marital status (%)				*χ*^2^(4) = 3.70, *p* = 0.448
*Single*	20%	10%	20%	
*Married/couple*	62%	71.5%	70%	
*Divorced/widowed*	18%	18.5%	10%	
Smokers (%)	22%	20.5%	24%	*χ*^2^(2) = 0.19, *p* = 0.911
Alcohol consumption (%)				*χ*^2^(6) = 7.45, *p* = 0.281
*No alcohol*	66%	62%	54%	
*1 to 5 units/week*	32%	32%	44%	
*6 to 10 units/week*	0%	6%	2%	
*>10 units/week*	12%	0%	0%	
Migraine (%)	46%	38.8%	18%	***χ*** ^**2**^ **(2) = 9.38, *p* = 0.009**

Highest education level completed according to the French education system; Level 1: before high school; Level 2: accomplished high school; Level 3: 2 years after high school; Level 4: Bachelor’s degree, Level 5: Master’s degree, Engineering degree, PhD, MD. ^*^Statistically significant differences are in bold.

### Procedures

Eligible participants were informed by otoneurologist ME about the study. Interested individuals were directed to the researchers and signed an informed consent. Then, participants answered demographic questions, the NPQ, DHI, HADS and STAI. At the beginning of the study, participants answered questionnaires on a tablet touch screen using PsychoPy® (23). Missing values due to technical difficulties led us to continue the data collection on printed paper questionnaires for the remaining participants. In total, 31 participants filled out paper questionnaires (15 PPPD patients, 3 vestibular patients and 13 healthy controls).

### Data analysis

Statistical analyses were carried out in IBM SPSS (version 25), GraphPad Prism (version 10.1.0) and JASP (version 0.16.1). We used two-tailed tests for all analyses and *p-values* were considered statistically significant when <0.05, unless otherwise specified.

#### Reliability analysis of the NPQ

The NPQ reliability was measured using Cronbach’s alpha (α). Reliability is considered good for a Cronbach’s α of 0.70–0.95, and poor for a Cronbach’s α below 0.70 (24).

#### Intergroup comparisons of NPQ, DHI, and HADS scores

As questionnaire ratings were not normally distributed, we used Kruskal–Wallis tests for between-group comparisons.

#### Effect of clinical parameters on NPQ scores

First, we stratified PPPD patients according to the history of vestibular disorder to control its effect on the NPQ total score with a Kruskal-Wallis test. Then, we conducted multiple linear regression analyses with clinical factors that may influence the NPQ scores (presence/absence of deficits at the caloric tests and the vHIT, presence/absence of migraine, and duration of the disease). The models met the assumptions for multiple linear regressions.

#### Effect of antidepressant treatment on NPQ scores

PPPD patients referred to our otoneurological center were included irrespective of the treatments taken at the time of inclusion, in line with procedures from Yagi et al. (9). Given the variability in the NPQ scores reported previously (9), we stratified PPPD patients and patients with vestibular disorders according to their antidepressant treatment at the time of inclusion and compared them using a Kruskal–Wallis test.

#### ROC curve analysis

We carried out receiver operating characteristics (ROC) curve analyses to determine how good the NPQ total score and sub-scores are to classify patients into PPPD and other vestibular disorders. We evaluated the area under the curve (AUC) as a measure of the accuracy of the classification. An AUC of 0.5 reflects results from a random classifier, whereas an AUC of 1 reflects the perfect sensitivity and specificity of the classifier. Discrimination is considered to have failed for an AUC between 0.5 and 0.6, to be poor for an AUC between 0.6 and 0.7, fair for an AUC between 0.7 and 0.8, good for an AUC between 0.8 and 0.9, and excellent for an AUC between 0.9 and 1 (25).

#### Relation between NPQ and reported handicap, depression, and anxiety

As questionnaire ratings were not normally distributed, we calculated non-parametric Spearman correlations between dependent variables.

## Results

### Sample characteristics

At the time of measurement, there was no statistical significance between the three groups of participants regarding their age, sex, handedness, education, employment status, marital status, smoking habits, and alcohol consumption (Table 1). As expected, the occurrence of migraine differed between the three groups [*χ*^2^_(2)_ = 9.38, *p* = 0.009], with the highest rate in PPPD patients (Table 1).

54% of the PPPD patients had a documented history of past or current vestibular disorder, including benign paroxysmal positional vertigo (*n* = 4), and/or vestibular migraine (*n* = 5), hydrops (*n* = 3), vestibular schwannoma (*n* = 2), semicircular canal dehiscence (*n* = 1), vestibular neuritis (*n* = 1), neurovascular conflict (*n* = 1), and/or other forms of unilateral or bilateral vestibulopathy (*n* = 10). Patients with other vestibular disorders presented with Menière’s disease (*n* = 15) and/or benign paroxysmal positional vertigo (*n* = 7), semicircular canal dehiscence (*n* = 3), hydrops (*n* = 2), other unilateral or bilateral vestibulopathy (*n* = 20), and/or vestibular migraine (*n* = 4).

Table 2 summarizes clinical parameters in the two groups of patients. While the occurrence of migraine did not differ significantly between PPPD patients and patients with vestibular disorders [*χ*^2^_(1)_ = 0.53, *p* = 0.727], the duration of the disease was significantly longer in patients with vestibular disorders than in patients with PPPD (Mann–Whitney test, *U* = 642, *p* < 0.01). A larger proportion of PPPD patients was under antidepressant medication at the time of inclusion in the study (18 were under SSRI), when compared to patients with vestibular disorders (3 under SSRI, 1 under monoamine oxidase inhibitor; *χ*^2^_(1)_ = 13.56, *p* < 0.001).

**Table 2:** Descriptive statistics of the clinical parameters.

Clinical parameters	PPPD	Vestibular	Statistics^*^
**Disease duration (months)**			
*n*	48	43	
*Mean ± SD*	74.55 ± 216.04	108.89 ± 221.63	
*Mdn*	25.5	65	***U* = 642, *p* = 0.002**
**Migraine**			
*Rate*	23/50	19/49	
*%*	46.0%	38.8%	*χ*^2^(1) = 0.53, *p* = 0.727
**Antidepressant treatment**			
*n*	18/50	4/50	
*%*	36.0%	6%	***χ*** ^**2**^ **(1) = 13.56, *p* < 0.001**
**Spontaneous, positional or HST-evoked nystagmus**			
*Rate*	24/45	35/45	
*%*	53.3%	77.8%	***χ*** ^**2**^ **(1) = 5.95, *p* = 0.015**
**Abnormal caloric test**			
*Rate*	4/15	20/29	
*%*	26.7%	69.0%	***χ*** ^**2**^ **(1) = 7.13, *p* = 0.008**
**Vestibular loss at caloric test (%)**			
*n*	15	29	
*Mean ± SD*	17.96 ± 32.08%	46.03 ± 33.32%	
*Mdn*	0	39	***U* = 100.5, *p* = 0.003**
**Abnormal vHIT**			
*Rate*	7/22	15/33	
*%*	31.8%	45.5%	*χ*^2^(1) = 1.02, *p* = 0.312
**vHIT left ear (gain)**			
*n*	22	32	
*Mean ± SD*	0.93 ± 0.21	0.84 ± 0.17	
*Mdn*	0.92	0.86	***U* = 236.5, *p* = 0.042**
**vHIT right ear (gain)**			
*n*	22	32	
*Mean ± SD*	0.95 ± 0.33	0.95 ± 0.18	
*Mdn*	0.96	0.96	*U* = 342, *p* = 0.865

Clinical assessment was in general not performed at the time participants filled out the questionnaires. Data were collected from the patient’s medical records. *Rate* indicates the number of positive findings relative to the number of patients in which the variable has been measured (*n*). Abnormal values for the caloric test are absolute values of unilateral weakness (i.e., percent canal paresis) > 25%. Unilateral weakness in % was calculated as the difference between the caloric response from the right and left ears by the Jongkees equation, where 0 indicates that the responses from the right and left ears are equal, and ± 100% indicate a total lack of caloric response from one ear. To simplify and allow between-groups comparisons, the absolute value of the unilateral weakness in % is reported. Abnormal values for the vHIT (lateral semicircular canals) are gains <0.8 or >1.2. Parameters were compared between groups using Mann–Whitney and Chi-Square tests. ^*^Statistically significant differences are in bold.

Although a comprehensive clinical assessment of vestibular functions was not conducted in all patients, the data available in the patients’ records suggest that patients with PPPD had lower than or similar vestibular deficits to patients with other vestibular disorders (Table 2). When analyzing the presence of a nystagmus throughout the patients’ history of the disease, we found that patients with PPPD have presented significantly less often one or several types of nystagmus (i.e., spontaneous, positional, or nystagmus evoked by the head shaking test) than patients with other vestibular disorders [*χ*^2^_(1)_ = 5.95, *p* < 0.05]. Results from the caloric tests showed that patients with PPPD have in the history of their disease shown a significantly lower percentage of unilateral weakness (*U* = 100.5, *p* < 0.05), and a lower rate of abnormal caloric tests (i.e., unilateral weakness >25%) than patients with other vestibular disorders [*χ*^2^_(1)_ = 7.13, *p* = 0.008]. Finally, although measured at various times in the history of their disease, patients with PPPD showed a significantly higher gain of the vHIT for the left lateral canal than vestibular patients (*U* = 236.5, *p* < 0.05), and both groups had similar vHIT gain for the right lateral canal (*U* = 342, *p* = 0.865). Overall, the rate of abnormal vHIT [i.e., gain <0.8 or >1.2 (26)] did not differ significantly in PPPD and vestibular patients [*χ*^2^_(1)_ = 1,02; *p* = 0.312].

### Reliability analysis of the NPQ

An internal consistency analysis was calculated for the clinical population, i.e., patients with PPPD and other vestibular disorders together (*n* = 100), and it revealed high reliability for all NPQ subscales (Upright/Walking: Cronbach’s α = 0.85; Movement: α = 0.81; Visual stimulation: α = 0.81). For the total NPQ score (12 items), Cronbach’s α was 0.92.

### Intergroup comparisons of NPQ scores

**Figure 2:**
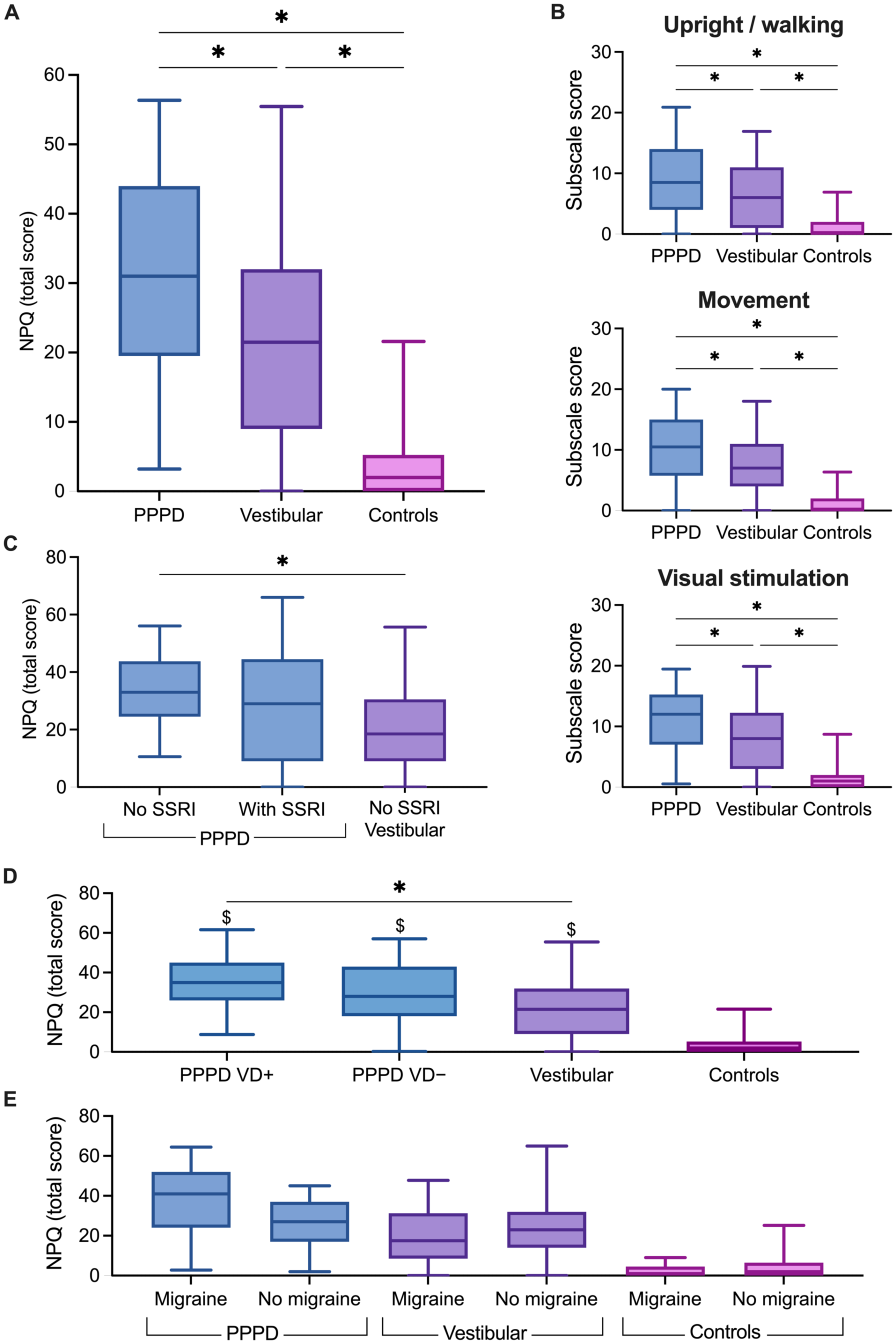
Niigata Persistent Postural-Perceptual Dizziness Questionnaire (NPQ) scores. Box plots showing **(A)** the total score, **(B)** the subscales score in the persistent postural-perceptual dizziness (PPPD), vestibular and control groups, **(C)** the total score in PPPD without selective serotonin reuptake inhibitors (SSRI), PPPD with SSRI and vestibular patients. **(D)** Total NPQ score in PPPD patients stratified according to a documented history of vestibular disorder (PPPD VD+) or no history of vestibular disorder (PPPD VD−). **(E)** Total NPQ score in participants stratified according to self-reported migraine. The solid line inside the box is the median and boxes cover the interquartile range (5–95). ^*^p < 0.05, Wilcoxon test. ^$^*p* < 0.05, Wilcoxon test with respect to the control group.

Figure 2A shows individual NPQ total scores for the three groups of participants. The NPQ total score was significantly modulated by the group (Kruskal–Wallis test, *H*(2) = 73.97, *p* < 0.001). As expected, pairwise comparisons with adjusted *p*-values showed significantly higher NPQ total scores in patients with PPPD (*Mdn* = 31) when compared to the controls (*Mdn* = 2; *p* < 0.001, *r* = 0.83) and to the patients with other vestibular disorders (*Mdn* = 21.5; *p* < 0.05, *r* = 0.22). Patients with other vestibular disorders also had significantly higher NPQ total scores than the controls (*p* < 0.001, *r* = 0.61). Consistently, NPQ sub-scores (Figure 2B) were significantly higher in PPPD patients than in patients with other vestibular disorders and in controls for the subscales Upright/Walking (PPPD vs. vestibular: *p* < 0.05, *r* = 0.22; PPPD vs. controls: *p* < 0.001, *r* = 0.72), Movement (*p* < 0.05, *r* = 0.20; *p* < 0.001, *r* = 0.81), and Visual stimulation (*p* < 0.05, *r* = 0.23; *p* < 0.001, *r* = 0.77).

### Effect of clinical parameters on NPQ scores

As most of our PPPD patients have a documented history of vestibular disorder, we explored the effect of stratification of the PPPD group into PPPD with (PPPD VD+) and PPPD without (PPPD VD−) a past or present vestibular disorder on the NPQ score (Figure 2D). The NPQ total score was significantly modulated by the group [Kruskal–Wallis test, *H*(3) = 75.15, *p* < 0.0001]. Pairwise comparisons with adjusted *p*-values showed no significant difference in the total NPQ scores in the PPPD VD+ patients (*Mdn* = 35) when compared to PPPD VD− patients (*Mdn* = 28; *p* = 0.28). The NPQ total score was significantly higher in PPPD VD+ patients than in patients with other vestibular disorders (*Mdn* = 21.5; *p* < 0.05). However, no significant difference was found in PPPD VD− patients when compared to patients with other vestibular disorders (*p* = 0.26). Both PPPD groups had significantly higher NPQ total scores than the control group (*Mdn* = 2; *p* < 0.0001).

Considering this effect of an history of vestibular disorder on the NPQ total scores, and the difference of PPPD patients and patients with other vestibular disorders on several clinical parameters (Table 2), we examined whether these parameters predicted NPQ scores. First, we calculated multiple linear regressions on the NPQ total scores separately for the subpopulation who underwent the vHIT and caloric tests, including group (PPPD vs. vestibular disorders) and vestibular deficit (as a yes/no variable) as predictors (Table 3). NPQ scores were not significantly predicted by the deficit at the vHIT (*t* = 0.439, *p* = 0.663) and the caloric tests for the lateral canals (*t* = 1.00, *p* = 0.322). Bearing in mind the small size of the subsamples, the effect of the group on the NPQ total score was significant when controlling for the effect of the deficit at the vHIT (*t* = −2.96, *p* = 0.005), and at a significance level when controlling for the effects of the deficit at the caloric test (*t* = −2.02, *p* = 0.05).

**Table 3:** Multiple linear regressions for the association of the NPQ scores with clinical parameters.

	***β* [95% CI]**	**SE**	***t***	***p***
**NPQ total score**				
*Group (vestibular)*	−13.530 [−22.696, −4.364]	4.568	−2.962	**0.005**
*vHIT lateral canal (normal)*	2.004 [−7.162, 11.169]	4.568	0.439	0.663
**NPQ total score**				
*Group (vestibular)*	−11.014 [−22.046, 0.018]	5.463	−2.016	0.050
*Caloric test (normal)*	5.211 [−5.291, 15.713]	5.200	1.002	0.322
**NPQ total score**				
*Disease duration*	0.006 [−0.008, 0.021]	0.007	0.876	0.384
*Group (vestibular)*	−8.906 [−15.210, −2.601]	3.172	−2.808	**0.006**
*Migraine (yes)*	6.321 [−0.030, 12.673]	3.195	1.978	0.051
**NPQ Upright/Walking score**				
*Disease duration*	0.006 [7.043 × 10^−4^, 0.012]	0.003	2.238	**0.028**
*Group (vestibular)*	−3.169 [−5.598, −0.741]	1.222	−2.594	**0.011**
*Migraine (yes)*	2.288 [−0.159, 4.735]	1.231	1.859	0.066
**NPQ Movement score**				
*Disease duration*	1.994 × 10^−4^ [−0.005, 0.006]	0.003	0.074	0.941
*Group (vestibular)*	−2.821 [−5.141, −0.501]	1.167	−2.417	**0.018**
*Migraine (yes)*	2.404 [0.067, 4.741]	1.176	2.044	**0.044**
**NPQ Visual simulation score**				
*Disease duration*	−1.004 × 10^−4^ [−0.006, 0.005]	0.003	−0.036	0.971
*Group (vestibular)*	−2.871 [−5.254, −0.488]	1.199	−2.394	**0.019**
*Migraine (yes)*	1.594 [−0.807, 3.995]	1.208	1.320	0.190

^*^Statistically significant differences are in bold. *β*, unstandardized coefficients; CI, confidence interval; SE, standard error. Note that linear regressions with results from the vHIT and caloric tests have been computed in a subsample of the patients in which clinical data are available (vHIT: 22 PPPD, 33 vestibular; caloric tests: 15 PPPD, 29 vestibular), whereas linear regressions with the factors migraine and duration of the disease have been computed on the whole patients sample.

Next, we assessed the effects of migraine and disease duration on the total NPQ scores. A multiple linear regression analysis (Table 3), including group, disease duration and migraine (as a yes/no variable) revealed that the group remained the only factor significantly associated with the NPQ total score while controlling for the effect of migraine and duration of the disease (*t* = −2.81, *p* = 0.006). Migraine was trending but not significant (*t* = 1.98, *p* = 0.051), and the duration of the disease was not a significant predictor of the NPQ total score (*t* = 0.88, *p* = 0.384). The group was also the main significant predictor of each NPQ subscale (Table 3) while controlling for the effect of migraine and duration of the disease (all *p* values < 0.05). Migraine was a significant predictor of the Movement score (*t* = 2.04, *p* = 0.044), but not of the other NPQ sub-scales, whereas duration of the disease was a significant predictor of the Upright/Walking score (*t* = 2.238, *p* = 0.028). To explore further the effect of migraine, participants from the three groups were stratified according to the occurrence of migraine (Figure 2E). Pairwise comparisons with adjusted *p*-values showed that NPQ total scores did not differ significantly between participants with and without migraine for the PPPD patients (*p* = 0.134), patients with vestibular disorders (*p* = 0.371) and controls (*p* = 0.723).

### Effect of antidepressant treatment on NPQ scores

When patients were stratified according to treatment, the NPQ total score was significantly modulated by the group [*H*(2) = 11.97, *p* < 0.01]. Pairwise comparisons with adjusted *p*-values showed that the NPQ total score did not differ between PPPD patients with SSRI (*Mdn* = 31) and without SSRI (*Mdn* = 31; *p* = 0.15, *r* = 0.15; Figure 2C). Interestingly, NPQ total scores were significantly higher in PPPD patients without SSRI than in patients with vestibular disorders without SSRI (*Mdn* = 18.5; *p* < 0.001, *r* = 0.35), whereas scores did not differ significantly between PPPD patients with SSRI and patients with vestibular disorders (*p* = 0.19, *r* = 0.13). No further analysis was run on the patients with vestibular disorders as only four of them were under antidepressant treatment.

### ROC curve analysis

**Figure 3:**
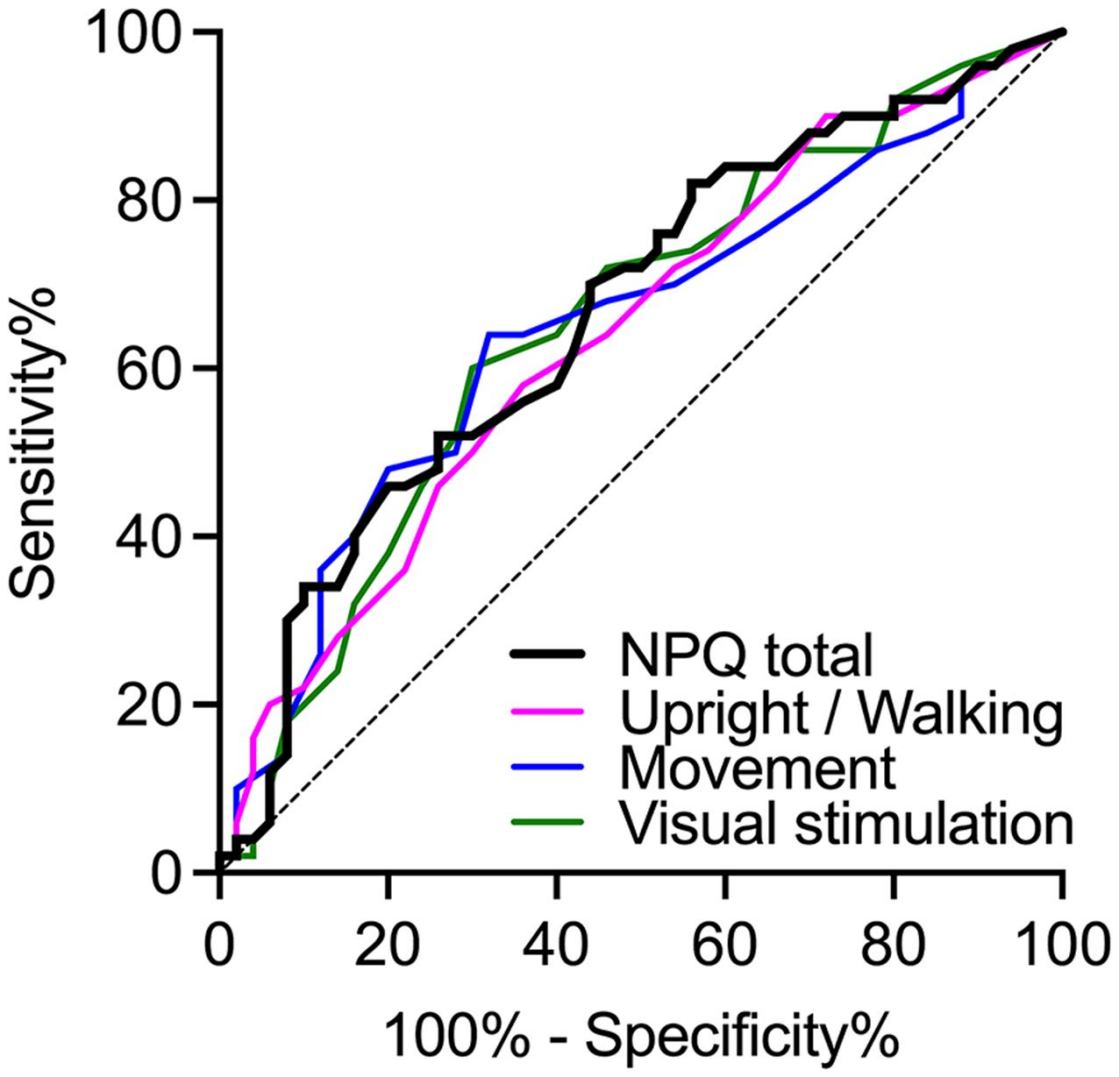
ROC analysis for detecting PPPD in patients with PPPD and vestibular disorders.

Next, we wanted to establish whether NPQ total score and sub-scores successfully discriminate between participants with PPPD and other vestibular disorders. The ROC curve analysis showed that NPQ scores successfully discriminate patients (Figure 3). NPQ total scores revealed a significant, but poor discrimination, with AUC = 0.664 (*p* = 0.005; 95% confidence interval (CI): 0.558–0.771) and a standard error of 0.054. Similarly, all NPQ subscales were significant but poor predictors of PPPD. The Upright/Walking subscale had an AUC = 0.638 (*p* = 0.017; 95% CI: 0.530–0.747), and a standard error of 0.055. The Movement subscale had an AUC = 0.648 (*p* = 0.011; 95% CI: 0.539–0.757), and a standard error of 0.056. The Visual stimulation subscale had an AUC = 0.652 (*p* = 0.009; 95% CI: 0.544–0.760), and a standard error of 0.055.

### Reported handicap, depression, and anxiety

**Figure 4:**
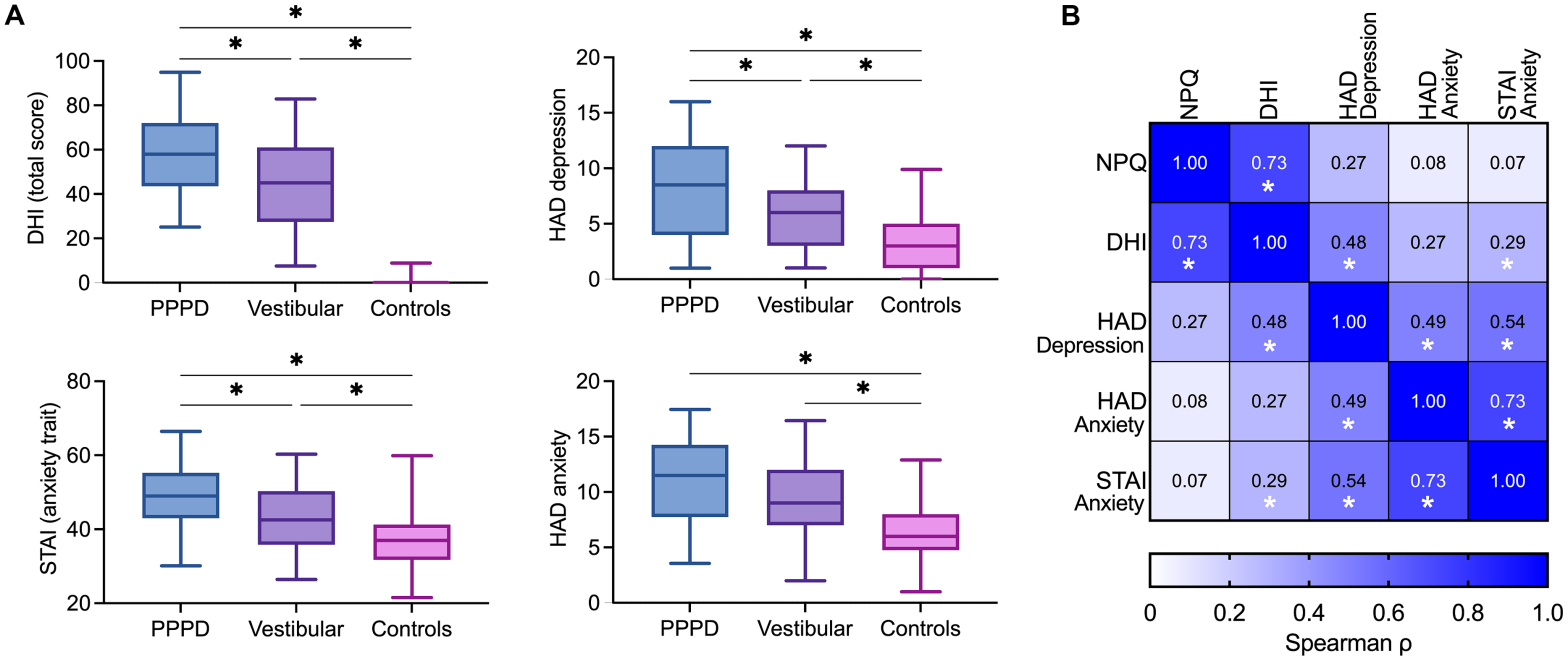
**(A)** Additional questionnaires. Box plots showing total scores of handicap related to dizziness (DHI), trait anxiety (STAI), depression (HADS-D) and state anxiety (HADS-A) in the PPPD, vestibular and control groups. The solid line inside the box is the median and boxes cover the interquartile range (5–95). ^*^p < 0.05, Wilcoxon test. **(B)** Correlation matrix between NPQ, DHI, HADS-D, HADS-A and STAI. Color intensity is proportional to correlation strength. ^*^p < 0.05, Spearman’s test.

Figure 4A shows the DHI and HADS scores for the three groups of participants. Consistent with the NPQ total score, DHI scores were significantly affected by the group [Kruskal–Wallis test, *H*(2) = 100.50, *p* < 0.001]. The median DHI scores were in the range of severe handicap for patients with PPPD (*Mdn* = 58), whereas it was in the range of moderate handicap for vestibular disorders (*Mdn* = 45). Pairwise comparisons with adjusted *p*-values showed that the DHI score was significantly higher in patients with PPPD when compared to controls (*Mdn* = 0; *p* < 0.001, *r* = 0.95) and patients with vestibular disorders (*Mdn* = 45; *p* < 0.05, *r* = 0.20). Patients with vestibular disorders also had significantly higher DHI scores than controls (*p* < 0.001, *r* = 0.75).

Depressive symptoms, measured by the HADS sub-score for depression, were significantly modulated by group [*H*(2) = 30.49, *p* < 0.001], with higher scores for patients with PPPD (*Mdn* = 8.5) than controls (*Mdn* = 3; *p* < 0.001, *r* = 0.55) and patients with vestibular disorders (*Mdn* = 6; *p* < 0.05, *r* = 0.21). Patients with vestibular disorders had significantly higher depressive symptoms than controls (*p* < 0.001, *r* = 0.34).

Results about the severity of anxiety differed for the state and trait of anxiety. The state of anxiety, measured by the HADS sub-score for anxiety, was significantly affected by group [*H*(2) = 25.76, *p* < 0.001]. Pairwise comparisons showed significantly higher *state* anxiety in patients with PPPD (*Mdn* = 11.5; *p* < 0.001, *r* = 0.49) and vestibular disorders (*Mdn* = 9; *p* < 0.001, *r* = 0.35) than in controls (*Mdn* = 6.5). State anxiety did not differ significantly between patients with PPPD and vestibular disorders (*p* = 0.187, *r* = 0.14). By contrast, the *trait* anxiety measured by the STAI [effect of group: *H*(2) = 27.17, *p* < 0.001] was significantly higher in PPPD patients (*Mdn* = 49) than in vestibular disorders (*Mdn* = 42.5; *p* < 0.01, *r* = 0.27) and controls (*Mdn* = 38; *p* < 0.001, *r* = 0.52).

### Relation between NPQ and reported handicap, depression, and anxiety

Figure 4B shows the matrix of nonparametric Spearman correlations between questionnaire scores in 50 patients with PPPD. NPQ total scores correlated significantly with DHI scores (*ρ* = 0.73, *p* < 0.001). However, there was no significant correlation between NPQ total scores and state of depression (*ρ* = 0.27, *p* = 0.06), state anxiety (*ρ* = 0.08, *p* = 0.60), or trait anxiety (*ρ* = 0.07, *p* = 0.61). By contrast, the DHI correlated significantly with state of depression (*ρ* = 0.48, *p* < 0.001), and trait anxiety (*ρ* = 0.29, *p* < 0.05), but not with state anxiety (*ρ* = 0.27, *p* = 0.06).

## Discussion

PPPD is a complex vestibular disorder and the NPQ is suggested to assist in PPPD diagnosis and assessment of symptom severity. Our study aimed to better understand the symptoms of patients with PPPD and to validate the NPQ in a Western French-speaking population. Our participant pool aligned with previous literature (1, 5, 6, 27) suggesting a higher percentage of women than men suffering from PPPD, an age range of 35–64 years and an average age of 49 years, close to the mean age of 53–59 years reported in previous studies in adult patients with PPPD [e.g., (28); reviewed in Staab (1)].

We firstly predicted that patients with PPPD would score higher on the average NPQ score and the subscale scores in comparison to the vestibular group and controls. The results strongly confirmed our predictions with the highest NPQ scores for the PPPD group, the second highest for the vestibular group, and the control group having the lowest NPQ scores, similar to Yagi et al. (9). Our ROC curve analysis showed that NPQ was a significant but poor predictor of PPPD, similar to a ROC curve analysis by Castillejos-Carrasco-Muñoz et al. (5), but poorer than the original analysis by Yagi et al. (9).

When we stratified PPPD patients to distinguish those with a history of vestibular disorder from those without, we found that the NPQ total score did not differ between the two groups. Nonetheless, our analysis indicated that only the PPPD patients with past or present vestibular disorders significantly differed from patients with other vestibular disorders. This might mean that a prior or current vestibular disorders can increase the severity of PPPD symptoms. However, some studies interestingly suggest that vestibular function in PPPD patients can be altered (even when no precipitating vestibular condition is identified) (29) and that vestibular dysfunction in PPPD patients is influenced by preceding balance disorders (30). Thus, PPPD seems intrinsically related to a certain degree of vestibular dysfunction, depending on the patients, and this might be part of the explanation for the severity of symptoms. Nonetheless, in our analysis of the influence of migraine, disease duration, and vestibular deficits on PPPD, we observed that the variance in the NPQ scores among patients with PPPD and patients with other vestibular disorders was not attributed to differences in the severity of vestibular deficits. As PPPD often co-exists with other vestibular disorders (1), it would be interesting for future studies to investigate the differential clinical characteristics of PPPD alone and PPPD with vestibular disorders and to assess the effect this differentiation can have on symptom severity and exacerbating factors.

Interestingly, despite PPPD patients exhibiting either lower or comparable levels of vestibular deficits compared to patients with other vestibular disorders and presenting with shorter disease durations (Table 2), the duration of PPPD did not emerge as a significant predictor of NPQ total scores, except for the Upright/Walking subscale. This finding contrasts with the observations by Yagi et al. (9), who reported no significant impact of disease duration on PPPD subtypes. Our results align better with the findings of Teh and Prepageran (27), demonstrating that disease duration may contribute to increased anxiety levels and physical handicap in PPPD patients, suggesting a potential nuanced relationship between disease duration and NPQ scores, even though disease duration only predicted Upright/Walking subscale.

As the NPQ measures the severity of symptoms when being exposed to optic flows and self-motion, scores may be influenced by additional migraine. NPQ scores did not differ in participants with and without migraine. In addition, our multiple linear regression models revealed a significant effect of the group on the NPQ scores when controlling for the effect of migraine. Migraine was trending but not a significant predictor for the NPQ total score, whereas migraine was a significant predictor only for the Movement NPQ sub-scale. This stronger self-reported sensibility to motion explained by migraine is in line with laboratory measures showing an increased sensitivity to head motion in patients with vestibular migraine (31, 32). Accordingly, the severity of the symptoms measured by the NPQ can be partly related to additional migraine, which is intrinsic to and more frequent in PPPD (2, 33, 34). Vestibular migraine was the trigger for 11–25% of PPPD cases in Staab (1) and was a common comorbidity in the recent review by Trinidade et al. (6). Powell et al. (13) researched PPPD as a spectrum in a sample from the general population and found that migraine correlated with PPPD but was not a strong predictor. Moreover, migraine is far more prevalent in women than men (35, 36), similar to PPPD. In conclusion, although migraine seems to potentiate symptoms from the Movement NPQ sub-scale, the NPQ total scores were better predicted by the group when statistically controlling for the effect of migraine.

Our second prediction that PPPD patients will differ significantly from other vestibular patients and controls on perceived handicap, depression and anxiety was supported by our findings. PPPD patients showed much higher levels of perceived handicap by their symptoms (DHI), depressive symptoms (HADS-depression) and trait anxiety (STAI-trait) than vestibular patients and controls. Because high levels of anxiety and depression are frequent comorbidities in vestibular patients (1, 2, 33, 37), they are not included as a separate PPPD diagnostic criterion (6), even though they severely impact the everyday life of PPPD patients (38). In addition, anxiety is a predisposing factor of PPPD, mainly in its trait form (1, 11), and contributes to its maintenance by anxiety-related responses to precipitating events, heightened body vigilance, and contribution to altered brain functional connectivity (1, 33, 39). Interestingly, in line with the results from Yagi et al. (9), we did not find a significant difference in the state anxiety score (HADS-anxiety) between vestibular and PPPD patients. The literature is still inconclusive, but these results direct to multifactorial high anxiety levels in vestibular disorders (40–43). In conclusion, PPPD seems to differ more from vestibular patients on *trait* anxiety than *state* anxiety.

Our correlation matrix showed a strong relationship between NPQ and DHI, but no other questionnaire. Given the similarities between the two questionnaires, future studies could evaluate the different factors assessed by each and potentially suggest the best way for practitioners to use them. In our study, we reported both NPQ and DHI scores to provide a holistic overview of our participants’ symptomatology, as other studies have done (1, 6, 10, 27). Future studies should aim to include more precise information to facilitate replicable results and provide a comprehensive overview of the patients.

Importantly antidepressant medication is now a commonly suggested treatment for PPPD with positive results, especially if combined with cognitive behavioral therapy, or vestibular rehabilitation (1). Our cross-sectional study, that recruited patients irrespective of treatment, does not allow for a formal investigation of the effect of antidepressant treatment on symptom severity, as a clinical trial would. In fact, we found no significant difference in NPQ scores between PPPD patients with and without treatment. We only observed a significant difference between PPPD and vestibular patients without antidepressant treatments, which might be related to the fact that patients on antidepressants may have experienced more severe PPPD symptoms before starting treatment. Future studies could benefit from (1) testing the same patient population before and after treatment for a more direct effect of treatment on everyday vestibular symptoms; (2) assessing the effect of medication dosage and (3) the differential and/or combined effects of antidepressants and other lines of treatment (including other medications, cognitive behavioral therapy, vestibular rehabilitation).

A recent study by Yagi et al. (44) suggested that there might be three subtypes of PPPD following the factors of NPQ: a visual-dominant type, an active motion-dominant type, and a mixed type. However, Castillejos-Carrasco-Muñoz et al. (5) failed to replicate this finding in a Western (Spanish) population. Behrendt et al. (12) aimed to translate and adapt the NPQ to the German population and resulted in including seven additional items to the original NPQ. Future studies should work together with clinicians and patients to identify if the NPQ is best used by them as a diagnostic tool, a measure of symptom severity, or both. Longitudinal studies should follow vestibular and PPPD patients through their disease trajectory to better identify potential predictors, the relationship of NPQ with other symptoms, and how different treatments are projected into patients’ perceived handicap and psychological characteristics.

One limitation of our study is that the clinical data were collected during the patients’ medical assessment and/or from the patients records in our otoneurological center. As a result, we do not have systematic clinical data for all our patients and sometimes lack information to characterize the vestibular hypofunction in both patients’ groups. Moreover, the assignment of vestibular and non-vestibular PPPD participants is not readily comprehensive. Consequently, we cannot rule out a confounding effect of vestibular hypofunction on the NPQ total and sub-scores in the PPPD group. Future studies should aim to systematically assess patients to make a clear distinction between patients groups and control the effect of vestibular dysfunction on the NPQ scores.

To the best of our knowledge, our study is the first to provide such a holistic analysis and overview of PPPD symptomatology and include a healthy control group in addition to the group of vestibular patients for comparison. Our results show that NPQ is a promising questionnaire to measure the severity of PPPD symptoms, that can be administered in subsequent doctor visits to track the disease course and potential treatment benefits.

## Data availability statement

The raw data supporting the conclusions of this article will be made available by the authors, without undue reservation.

## Ethics statement

The studies involving humans were approved by CPP Île de France 2, n° 2021-A03111-40. The studies were conducted in accordance with the local legislation and institutional requirements. The participants provided their written informed consent to participate in this study.

## Author contributions

VM: Investigation, Software, Writing – original draft, Writing – review & editing. MG: Formal analysis, Investigation, Methodology, Writing – original draft, Writing – review & editing. JL: Investigation, Methodology, Visualization, Writing – review & editing. ME: Conceptualization, Investigation, Methodology, Project administration, Supervision, Validation, Visualization, Writing – review & editing. CL: Conceptualization, Formal analysis, Funding acquisition, Methodology, Project administration, Supervision, Validation, Writing – original draft, Writing – review & editing.
