# Supplementary material for: French adaptation and validation of the Niigata PPPD Questionnaire: measure of severity of Persistent Postural-Perceptual Dizziness and its association with psychiatric comorbidities and perceived handicap
Source: Front Neurol. 2024 Jul 30;15:1388805. doi: 10.3389/fneur.2024.1388805 (PMC11319117; doi:10.3389/fneur.2024.1388805)
Supplement: Supplementary file 1 [file Data_Sheet_1.PDF]

Supplementary Table 1. Steps for the French translation and cross-cultural adaptation of the Niigata PPPD questionnaire.

	**ORIGINAL QUESTIONNAIRE Niigata PPPD questionnaire from Yagi et al. (2019)**	**STEP 1: FORWARD TRANSLATION Translation of the questionnaire into French**	**STEP 2: BACK TRANSLATION Back translation of the questionnaire into English**	**STEP 3: FINAL VERSION Consensus translation of the questionnaire into French**
Instructions	The purpose of this questionnaire is to identify the difficulties in daily life activities that you may be experiencing due to dizziness.<br>Please indicate your answer by circling the number that best describes the extent to which you have been affected during the past week.<br>If you completely avoid performing any of these actions, circle the number 6.	Le but de ce questionnaire est d'identifier les difficultés dans les activités de la vie quotidienne que vous pouvez rencontrer en raison de vos vertiges et instabilités.<br>Veuillez répondre en entourant le chiffre qui décrit le mieux à quel point vous avez été gêné(e) au cours de la semaine écoulée.<br>Si vous évitez complètement d'effectuer l'une ces actions, entourez le chiffre 6.	The aim of this questionnaire is to identify the difficulties during daily activities that you can encounter due to your dizziness and instability. Kindly answer by encircling the number, which best describes to what degree you have been ill at ease during the past week.<br>If you completely avoid carrying out one of these actions, encircle number 6.	Le but de ce questionnaire est d'identifier les difficultés dans les activités de la vie quotidienne que vous pourriez rencontrer en raison de vos vertiges et instabilités.<br>Veuillez répondre en entourant le chiffre qui décrit le mieux à quel point vous avez été gêné(e) au cours de la semaine écoulée.<br>Si vous évitez complètement d'effectuer l'une ces actions, entourez le chiffre 6.
Q1	Quick movements such as standing up or turning your head	Mouvements rapides tels que se lever ou tourner votre tête	Quick movements such as getting up or turning your head	Faire des mouvements rapides tels que se lever ou tourner sa tête
Q2	Looking at large store displays	Regarder de grands rayonnages dans les magasins	Looking at big sets of shelves in stores	Regarder de grands rayonnages dans les magasins
Q3	Walking at a natural pace	Marcher à allure normale	Walking at a normal speed	Marcher à allure normale
Q4	Watching TV or movies with intense movement	Regarder la télévision ou des films contenant des mouvements intenses	Watching the tv or films containing intense movements	Regarder la télévision ou des films contenant des mouvements rapides
Q5	Riding a car, bus, or train	Prendre la voiture, le bus ou le train	Taking the car, the bus or the train	Voyager en voiture, bus ou train
Q6	Sitting upright in a seat without back and arm support	Être assis en position verticale sur un siège sans dossier ni accoudoir	Sitting in a vertical position on a seat with no back rest or arms.	Être assis sur un siège sans support pour le dos et les bras
Q7	Standing without touching fixed objects	Se tenir debout sans toucher d'objets fixes	Standing without touching anything	Se tenir debout sans toucher quelque chose de fixe
Q8	Watching a scroll screen on a PC or smartphone	Regarder un écran qui défile sur un ordinateur ou un Smartphone	Watching a screen which scrolls (down or across) on a computer or a Smartphone	Regarder un écran qui défile sur un ordinateur ou un Smartphone
Q9	Performing activities such as housework or light exercise	Effectuer des activités telles que des travaux ménagers ou des exercices légers	Carrying out activities such as housework or light exercises	Effectuer des activités telles que des travaux ménagers ou des exercices légers
Q10	Reading small letters in a book or newspaper	Lire de petites lettres dans un livre ou un journal	Reading small letters in a book or newspaper	Lire de petits caractères dans un livre ou un journal
Q11	Striding at a rapid pace	Marcher rapidement à grandes enjambées	Walking quickly with large strides	Marcher rapidement à grandes enjambées
Q12	Riding an elevator or escalator	Prendre un ascenseur ou un escalator	Taking a lift or escalator	Prendre un ascenseur ou un escalator
