# Supplementary material for: French adaptation and validation of the Niigata PPPD Questionnaire: measure of severity of Persistent Postural-Perceptual Dizziness and its association with psychiatric comorbidities and perceived handicap
Source: Front Neurol. 2024 Jul 30;15:1388805. doi: 10.3389/fneur.2024.1388805 (PMC11319117; doi:10.3389/fneur.2024.1388805)
Supplement: Supplementary file 2 [file Data_Sheet_2.PDF]

Niigata PPPD questionnaire

Date : ___________________

Le but de ce questionnaire est d'identifier les problèmes que vous pourriez avoir dans les activités de la vie quotidienne à cause de vos vertiges et instabilités.
Veuillez répondre en entourant le nombre qui décrit à quel point vous avez été gêné **au cours de la dernière semaine**.

Si vous avez complètement évité de réaliser ces actions, entourez le nombre 6.

		Aucun problème						Insupportable
Q1	**Mouvements rapides comme se lever ou tourner votre tête**	0	1	2	3	4	5	6
Q2	**Regarder de grands rayonnages dans les magasins**	0	1	2	3	4	5	6
Q3	**Marcher à allure normale**	0	1	2	3	4	5	6
Q4	**Regarder la télé ou des films contenant des mouvements intenses**	0	1	2	3	4	5	6
Q5	**Prendre la voiture, le bus ou le train**	0	1	2	3	4	5	6
Q6	**Être assis droit sur un siège sans dossier ni accoudoir**	0	1	2	3	4	5	6
Q7	**Se tenir debout sans toucher d'objets stables**	0	1	2	3	4	5	6
Q8	**Regarder un écran qui défile sur un ordinateur ou un Smartphone**	0	1	2	3	4	5	6
Q9	**Faire des activités comme le ménage ou des exercices peu intenses**	0	1	2	3	4	5	6
Q10	**Lire de petites lettres dans un livre ou un journal**	0	1	2	3	4	5	6
Q11	**Marcher rapidement à grandes enjambées**	0	1	2	3	4	5	6
Q12	**Prendre un ascenseur ou un escalator**	0	1	2	3	4	5	6
